# Microwave-assisted Phospha-Michael addition reactions in the 13α-oestrone series and *in vitro* antiproliferative properties

**DOI:** 10.1080/14756366.2021.1963241

**Published:** 2021-08-27

**Authors:** Erzsébet Mernyák, Sándor Bartha, Lili Kóczán, Rebeka Jójárt, Vivien Resch, Gábor Paragi, Máté Vágvölgyi, Attila Hunyadi, Bella Bruszel, István Zupkó, Renáta Minorics

**Affiliations:** a Department of Organic Chemistry, University of Szeged, Szeged, Hungary; b Department of Pharmacodynamics and Biopharmacy, University of Szeged, Szeged, Hungary; c Department of Medicinal Chemistry, University of Szeged, Szeged, Hungary; d MTA-SZTE Biomimetic Systems Research Group, University of Szeged, Szeged, Hungary; e Institute of Physics, University of Pécs, Pécs, Hungary; f Department of Pharmacognosy, University of Szeged, Szeged, Hungary

**Keywords:** Phospha-Michael addition, 13α-oestrone, α,β-unsaturated ketone, antiproliferative effect, tumour selectivity

## Abstract

Microwave-assisted phospha-Michael addition reactions were carried out in the 13α-oestrone series. The exocyclic 16-methylene-17-ketones as α,β-unsaturated ketones were reacted with secondary phosphine oxides as nucleophilic partners. The addition reactions furnished the two tertiary phosphine oxide diastereomers in high yields. The main product was the 16α-isomer. The antiproliferative activities of the newly synthesised organophosphorus compounds against a panel of nine human cancer cell lines were investigated by means of MTT assays. The most potent compound, the diphenylphosphine oxide derivative in the 3-*O*-methyl-13α-oestrone series (**9**), exerted selective cell growth-inhibitory activity against UPCI-SCC-131 and T47D cell lines with low micromolar IC_50_ values. Moreover, it displayed good tumour selectivity property determined against non-cancerous mouse fibroblast cells.

## 1. Introduction

Organophosphorus derivatives (OPs) represent an extensive class of organic compounds with diverse biological activities^1^. They have been widely applied in medicine^2^^,^^3^, agriculture^4^ and industry^5^ among others. Osteoporosis is one of the most frequent diseases in the world^6^. The treatment of osteoporosis is mostly based on bisphosphonates, owing to their multiple beneficial activities^7^. Their high affinity for calcium allows to target bone mineral selectively. They substantially inhibit tumour-induced bone destruction, tumour angiogenesis, and induce apoptosis in tumour cells. Certain OPs have found their application as anticancer agents^8^^,^^9^. Their mechanism of action relies on their alkylating ability. Cyclophosphamide and ifosfamide are currently used for the treatment of several bone and soft tissue sarcomas^10^. Combretastatin A-4 phosphate is a dual-action anticancer agent in clinical trials, having microtubule destabilising and vascular targeting properties^11^. Phosphate or thiophosphate esters of coumarin or flavone derivatives have found their application against hormone-dependent breast cancers^12^^,^^13^. These compounds, owing to their steroid sulfatase (STS) inhibitory activity, might suppress oestrogen biosynthesis in the mammary glands. The development of these potential drug candidates was based on replacement of the sulphate group of sulfatase inhibitors with mimics such as phosphate or thiophosphate. A similar strategy seemed to be useful in the design of steroid 5α-reductase inhibitors, too. 3-Phosphinic acid derivatives of certain steroids displayed nanomolar K_i_ values^14^.

Literature reveals the existence of OPs of natural compounds, including those of steroids. Natural oestrone has a wide range of applications in the development of potent enzyme inhibitors and anticancer agents^15–17^. However, the small set of synthetic oestrone-based OPs is mainly limited to compounds functionalised at the D- and/or the A-ring. Palladium-catalysed cross coupling reactions facilitated the synthesis of derivatives phosphorylated at the A-ring^18–20^. Organophosphorus oestrone derivatives substituted at C-17 or C-17a or fused to the D-ring are also known, but their biological activities are unexplored^21–25^. This might be due to their retained oestrogenic action, which restricts their pharmacological application^26^^,^^27^. The hormonal activity might significantly be suppressed by the epimerisation of C-13 of natural oestrone^28^. The conformational change in 13α-oestrone and its 17-hydroxy counterparts results in the loss of oestrogenic activity^29^. However, a number of 13α-oestrone derivatives possess other important biological activities. We have recently published our findings with respect to enzyme inhibitory and antiproliferative potential of certain 13α-oestrone derivatives^30–38^. A number of D-ring-modified 13α-oestrone derivatives were shown to exert substantial inhibitory action on the growth of human cancer cell lines of gynaecological origin. Derivatives modified at C-3-*O* and/or C-16 should be highlighted concerning their outstanding cell growth-inhibitory properties with important structure–activity relationships^32^^,^^33^^,^^37^^,^. Consequently, development of additional 13α-oestrone derivatives with potential antiproliferative activities would be of particular interest.

The phospha-Michael addition is an important tool for the synthesis of OPs^39–42^. This P–C bond forming reaction is usually accomplished by the addition of > P(O)H species to α,β-unsaturated carbonyl compounds. The resulting OPs possess potential bioactivities^1^. The addition is usually carried out under basic conditions. However, application of a base might be omitted. Literature describes even solvent- and/or catalyst-free thermal or microwave-assisted phospha-Michael reactions^41^^,^^42^^,^. The latter simple, but efficient strategy facilitates the convenient late-stage modification of biomolecules.

Having developed an experience in microwave-assisted steroid synthesis^31^^,^^38^, here we report the synthesis of 13α-oestrone derivatives phosphorylated at the D-ring, as potential anticancer agents. Microwave-assisted phospha-Michael addition reactions were planned, starting from exocyclic 16-methylene-17-ketones as α,β-unsaturated carbonyl compounds. Secondary phosphine oxides bearing different aryl substituents were used as nucleophilic partners. Our aim was to determine the antiproliferative properties of the newly synthesised γ-ketophosphine oxides against a panel of human cancer cell lines.

## 2. Materials and methods

Chemical syntheses, characterisation data of the reported compounds and selected 2 D NMR spectra, as well as experimental conditions of antiproliferative assays performed are described in the Supporting Information. Computational details are also explained in the Supporting Information.

## 3. Results and discussion

### 3.1. Chemistry

**Scheme 1.**
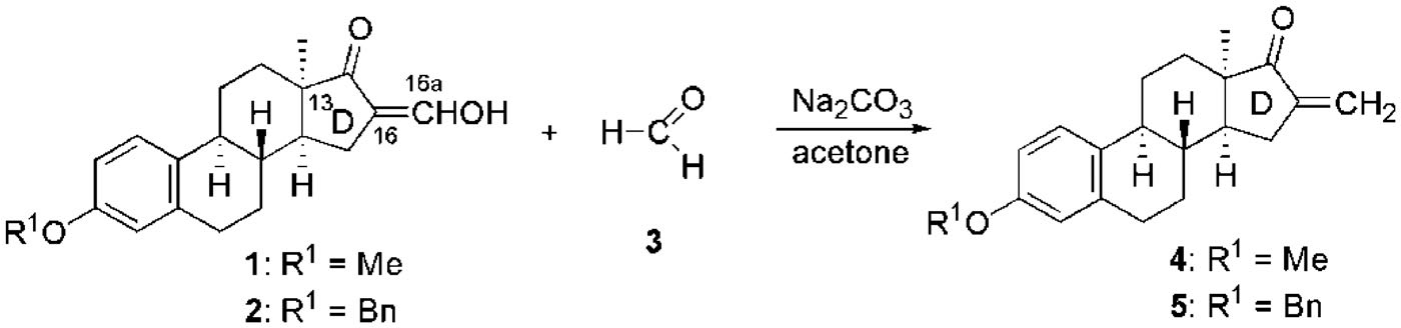
Synthesis of 16-methylene-13α-oestrone derivatives (**4** and **5**).

The efficient one-step synthesis of the known steroidal α,β-unsaturated ketones **4** or **5** was carried out from 16-hydroxymethylidene derivatives **1** or **2**, using formalin as a reagent and sodium carbonate as a base (Scheme 1)^43^^,^^44^. The resulting 3-benzyloxy- and 3-methoxy-16-methylene compounds (**4** and **5**) served as starting materials in the phospha-Michael addition reactions.

**Scheme 2.**
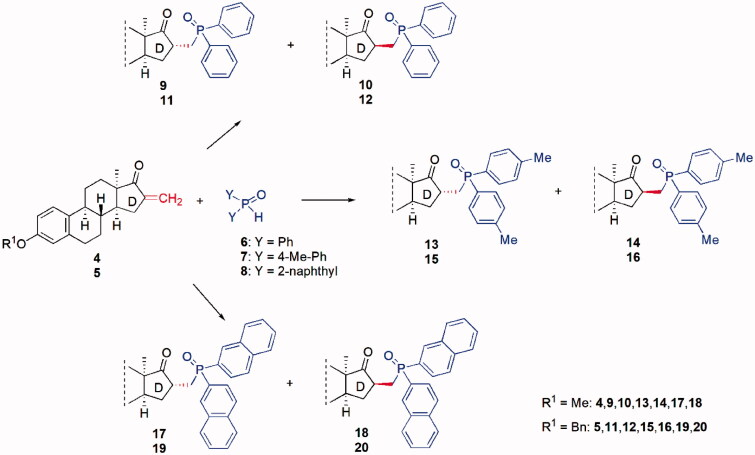
Phospha-Michael addition reactions in the 13α-oestrone series.

In our first attempt, diphenylphosphine oxide (**6**) was reacted with 3-methoxy-16-methylene derivative **4** in acetonitrile as solvent. The mixture was irradiated in a microwave reactor at 100 °C for 60 min without addition of a base. Thin layer chromatography indicated full conversion of the starting material and the formation of two reaction products. The attack of the P-nucleophile at C-16a resulted in the formation of phosphorylated 16α- and 16β-diastereomers. The 16α:16β = 2.3:1 diastereomeric ratio was established from the ^1^H NMR spectrum of the crude product, containing solely the two diastereomers. After microwave irradiation, the reaction mixture was allowed to cool to room temperature. The majority of the 16α-isomer (**9**) was obtained in pure form as a white precipitate. The solid was filtered, and the solvent was removed from the filtrate by evaporation *in vacuo*. The remaining diastereomeric mixture from the filtrate was separated by flash chromatography and/or preparative RP-HPLC using a Phenomenex Biphenyl column. After evaporation of the solvent minor diastereomer **10** was isolated as white crystals. The simple microwave-assisted synthetic methodology elaborated for the reaction of compound **4** with diphenylphosphine oxide (**6**) was extended to the transformations depicted in Scheme 2. The reaction time and temperature were varied as indicated in Table 1, according to the nature of the P(*O*)H reagent and the 3-*O*-substituent. Transformations of the 3-methyl ether (**4**, Table 1, Entries 1–3) occurred at lower temperatures compared to those of the 3-benzyl ether (**5**, Table 1, Entries 4–6). Reactions utilising (di-*para*-tolyl)phosphine oxide **7** as nucleophilic partner required longer reaction times at the same temperatures (Table 1, Entries 2 and 5) than those of reagent **6** (Table 1, Entries 1 and 4). Additions with the di(naphthalen-2-yl)phosphine oxide reagent (**8**) required elevated reaction temperatures (Table 1, Entries 3 and 6). The different reaction conditions essential for the completion of the addition reactions might presumably be attributed to phosphine **6**–**8** having different reactivity and the steric hindrance caused by the naphthyl groups. However, the ratio of the two diastereomers can be considered nearly the same, irrespective of the nature of the phosphine oxide substituents.

**Table 1.** Phospha-Michael addition reactions of α,β-unsaturated ketones (**4** or **5**) with secondary phosphine oxides (**6**, **7,** or **8**)^a,b^.

Entry	Substrate	Reaction time (min)	Temp (°C)	16α-isomer	16β-isomer	Yield^c^ (%)
1	**4**	60	100	**9**	**10**	87
2	**4**	90	100	**13**	**14**	90
3	**4**	60	130	**17**	**18**	92
4	**5**	60	125	**11**	**12**	91
5	**5**	90	125	**15**	**16**	89
6	**5**	60	140	**19**	**20**	87

^a^Reagents and conditions: α,β-unsaturated ketone (**4** or **5**, 1 equiv), secondary phosphine oxide (**6**, **7** or **8**, 1 equiv), acetonitrile.

^b^Ratio (16α:16β = 2.3:1) obtained from the ^1^H NMR spectrum of the crude diastereomeric mixture.

^c^Combined yields of the two diastereomers obtained after flash chromatography.

The structures of the newly synthesised γ-ketophosphine oxides were confirmed by ^1^H and ^13 ^C one- and two-dimensional NMR measurements (COSY, NOESY, HSQC and HMBC). The orientation of 16-H was deduced from the NOESY spectrum of compound **20** (Supporting material, Figure S2). A crosspeak was observed between the signals of 16-H and 13-Me, referring to α-orientation of 16-H.

### 3.2. Antiproliferative activities

**Figure 1.**
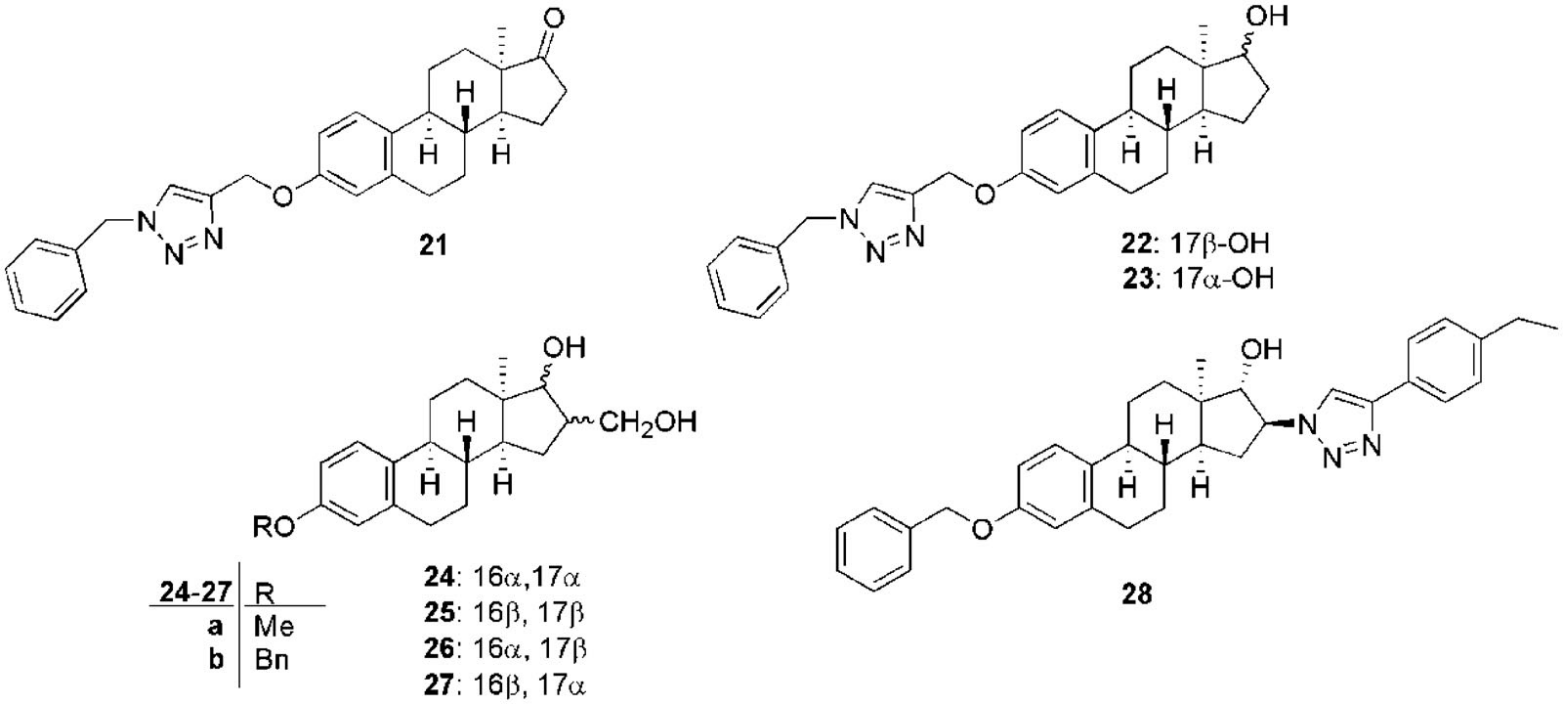
Structures of the recently described antiproliferative 13α-oestrone derivatives (**21**‒**28**).

We have recently described the development of a number of potential anticancer compounds based on the hormonally inactive 13α-oestrane core^32–34^^,^^37^^,^^45^^,^^46^. Modifications at C-3 influenced the cytostatic properties markedly. Introduction of a triazole moiety seemed to be highly advantageous^33^^,^^46^. The 3-[{1-benzyl-1*H*-1,2,3-triazol-4-yl}methoxy]-13α-oestrone derivative (**21**) displayed substantial antiproliferative action against human cancer cell lines of gynaecological origin, with IC_50_ values in the range of 0.3‒0.9 μM (Figure 1)^33^. However, the high cytostatic potential was associated with low cell-line selectivity. The epimeric 17-hydroxy counterparts of triazoles **22** and **23** exerted activities similar to that of the 17-ketone^33^. Consequently, the configuration of C-17 did not have marked influence on the cell growth-inhibitory properties. In addition, transformations at C-16 of the 17-hydroxy 3-ether derivatives of 13α-oestrone were performed^37^. The 16-hydroxymethylene (**24**‒**27**)^37^ and the 16-phenyltriazolyl derivatives (**28**)^32^ suppressed the growth of certain cancer cell lines with IC_50_ values in the low micromolar range (Figure 1). The orientation of the 16 and 17 substituents influenced the antiproliferative properties. The presence of the 3-benzyl ether moiety proved to be advantageous concerning the cell growth-inhibitory action. The inhibitory data obtained for 16β-hydroxymethylene-17α-hydroxy (**27 b**) and 16β-phenyltriazolyl-17α-hydroxy (**28**) compounds reveal that the nature of the C-16 substituent strongly influences both the effectiveness and the selectivity. One of the most potent compounds was triazole **28**, which induced cell cycle blockade at the G2–M transition and apoptosis *via* the intrinsic pathway^32^. The results mentioned above suggest that the development of potential anticancer compounds through modification of the 13α-oestrane core at C-3, C-16 and C-17 might provide important structure–activity information for the design of more active and selective cytostatic agents.

Here we evaluated the *in vitro* antiproliferative capacity of twelve newly synthesised, 16-substituted, 13α-oestrane-based γ-ketophosphine oxides (**9**‒**20**) and their precursors (**4** and **5**) on a panel of human adherent cancer cell lines. The compounds were tested against breast (MCF-7, MDA-MB-231 and T47D), ovarian (A2780), cervical (HeLa, SiHa and C33-A) and oropharyngeal (UPCI-SCC-131 and UPCI-SCC-154) carcinoma cell lines. Additionally, their tumour selectivity was also determined by using non-cancerous mouse embryo fibroblast (NIH/3T3) cells.

Our test compounds originate from 13α-oestrone 3-methyl and 3-benzyl ethers (**1** and **2**) bearing an exocyclic 16-methylene group (**4** and **5**). Based on their calculated IC_50_ values determined on all tested cancer cell lines, these parent substances can be considered as highly effective antiproliferative agents (IC_50_ = 2.0‒7.0 µM) (Table 2). On most cancer cell lines these cell growth-inhibitory activities are comparable to the antiproliferative effect of our positive control cisplatin, except HeLa, SiHa and MDA-MB-231 cell lines where **4** and **5** have IC_50_ values 2–5 times lower. On the other hand, these compounds inhibit cell division of non-cancerous cells in the same concentration range like of cancerous cells. Therefore, they can be considered as non-tumour selective compounds, which is not beneficial in the view of future development as drug candidates.

**Table 2.** Antiproliferative properties of the newly synthesised compounds

Comp.	Conc. (μM)	Growth inhibition; % ± SEM [calculated IC_50_ value; μM]^a^									
		UPCI-SCC-131	UPCI-SCC-154	HeLa	SiHa	C33-A	A2780	MCF-7	MDA-MB-231	T47D	NIH/3T3
4	10	99.80 ± 0.36	97.29 ± 1.10	85.11 ± 2.44	97.44 ± 0.54	99.57 ± 0.61	96.90 ± 1.52	99.52 ± 0.51	99.97 ± 0.71	99.68 ± 0.74	101.1 ± 0.67
	30	99.88 ± 0.39	97.81 ± 0.65	99.43 ± 0.28	99.28 ± 0.42	99.55 ± 0.39	100.5 ± 0.22	99.83 ± 0.44	94.64 ± 2.26	99.92 ± 0.71	100.9 ± 0.71
		[3.17]^b^	[5.15]	[4.45]	[3.31]	[3.60]	[6.24]	[3.70]	[3.97]	[3.46]	[2.79]
5	10	99.54 ± 0.33	96.40 ± 0.89	75.07 ± 3.73	98.98 ± 0.26	93.88 ± 2.56	94.85 ± 2.13	99.70 ± 0.43	97.15 ± 1.46	100.2 ± 0.28	100.8 ± 0.16
	30	99.94 ± 0.44	100.3 ± 0.82	99.65 ± 0.25	99.97 ± 0.42	100.0 ± 0.23	100.9 ± 0.18	100.6 ± 0.31	98.57 ± 0.89	101.3 ± 0.47	100.3 ± 0.25
		[2.38]	[4.50]	[6.99]	[2.30]	[3.75]	[6.70]	[3.35]	[4.07]	[3.47]	[2.74]
9	10	63.42 ± 1.41	–^c^	–	–	–	47.23 ± 2.75	21.29 ± 2.79	–	57.45 ± 3.15	–
	30	99.35 ± 0.27	99.82 ± 1.48	97.59 ± 0.73	96.49 ± 1.24	97.85 ± 0.32	98.99 ± 0.41	93.08 ± 1.60	99.03 ± 0.73	93.77 ± 0.55	96.55 ± 0.86
		[5.30]	[13.49]	[12.90]	[13.79]	[13.32]	[11.74]	[13.67]	[23.49]	[7.20]	[20.44]
13	10	29.88 ± 1.21	–	–	–	–	–	–	–	26.68 ± 1.95	–
	30	97.02 ± 0.43	75.94 ± 1.93	95.69 ± 0.69	92.90 ± 0.85	96.31 ± 0.46	97.77 ± 0.35	84.92 ± 2.54	95.56 ± 0.67	89.82 ± 0.68	89.76 ± 0.49
		[12.28]	[21.51]	[14.34]	[13.72]	[14.51]	[23.75]	[14.75]	[25.81]	[13.58]	[19.27]
17	10	26.48 ± 1.70	–	–	–	–	–	–	–	–	n. d.^d^
	30	45.31 ± 1.84	20.26 ± 2.69	–	–	–	–	–	–	27.12 ± 2.93	
11	10	20.86 ± 1.67	–	–	–	–	–	–	–	24.70 ± 1.70	n. d.
	30	62.45 ± 1.40	38.56 ± 2.60	23.80 ± 2.11	30.22 ± 2.99	40.26 ± 0.95	21.91 ± 2.63	37.06 ± 0.72	–	58.96 ± 1.38	
15	10	24.60 ± 1.39	–	–	–	–	–	–	–	39.07 ± 2.01	n. d.
	30	60.22 ± 1.32	34.66 ± 1.36	27.44 ± 1.57	–	26.94 ± 0.84	49.39 ± 2.06	–	–	58.50 ± 0.80	
19	10	–	34.27 ± 2.80	30.96 ± 1.11	23.24 ± 1.36	28.08 ± 1.39	–	31.84 ± 2.47	–	59.44 ± 2.44	n. d.
	30	40.98 ± 2.94	48.81 ± 2.15	48.89 ± 0.90	40.30 ± 2.00	34.69 ± 1.70	28.51 ± 2.35	40.60 ± 1.71	–	62.41 ± 1.70	
10	10	39.82 ± 1.36	–	–	–	21.96 ± 1.84	–	–	–	–	n. d.
	30	72.93 ± 1.70	24.32 ± 2.84	21.53 ± 2.99	–	27.25 ± 1.85	64.25 ± 2.65	35.85 ± 3.30	–	61.09 ± 1.06	
14	10	48.75 ± 1.50	–	–	–	26.86 ± 2.13	–	–	–	22.60 ± 1.33	–
	30	97.29 ± 1.38	87.05 ± 1.16	99.14 ± 0.37	92.97 ± 1.37	101.1 ± 0.24	100.3 ± 0.22	89.98 ± 2.88	98.22 ± 0.39	85.98 ± 1.51	52.71 ± 2.87
		[10.92]	[11.38]	[12.22]	[11.75]	[10.93]	[21.46]	[14.37]	[17.59]	[13.85]	[29.64]
18	10	46.39 ± 1.19	–	–	–	–	–	–	–	–	n. d.
	30	64.55 ± 1.29	–	–	–	20.25 ± 1.01	–	–	–	–	
12	10	49.38 ± 2.06	–	–	–	–	–	–	–	31.38 ± 1.55	–
	30	90.01 ± 1.15	75.46 ± 1.84	96.39 ± 1.88	94.83 ± 1.48	97.68 ± 0.85	97.14 ± 0.55	90.19 ± 1.61	84.01 ± 2.61	75.02 ± 1.65	48.34 ± 1.65
		[14.18]	[29.83]	[13.62]	[14.12]	[13.63]	[28.46]	[16.42]	[24.46]	[16.97]	[30.85]
16	10	49.45 ± 2.27	–	–	–	–	–	–	–	23.63 ± 1.22	n. d.
	30	67.44 ± 1.11	31.55 ± 2.41	31.83 ± 2.27	–	59.09 ± 0.69	67.27 ± 3.04	–	–	64.48 ± 1.60	
20	10	48.25 ± 1.46	30.97 ± 2.54	–	–	27.94 ± 0.89	–	28.24 ± 1.66	–	66.09 ± 0.76	n. d.
	30	61.12 ± 1.32	28.72 ± 1.11	–	–	26.53 ± 1.06	–	22.21 ± 2.78	–	68.56 ± 1.81	
CIS^e^	10	95.63 ± 1.49	87.40 ± 1.72	42.61 ± 2.33	88.64 ± 0.50	85.98 ± 1.05	83.6 ± 1.2	66.91 ± 1.81	–	40.41 ± 1.25	76.74 ± 1.26
	30	95.09 ± 1.57	92.72 ± 1.67	99.93 ± 0.26	90.18 ± 1.78	98.66 ± 0.21	95.0 ± 0.3	96.80 ± 0.35	71.47 ± 1.20	56.84 ± 1.16	96.90 ± 0.25
		[1.22]	[1.29]	[12.43]	[7.84]	[4.13]	[1.30]	[5.78]	[19.13]	[19.24]	[4.73]

^a^Mean value from two independent measurements with five parallel wells; standard deviation <20%.

^b^IC_50_ values have been calculated if the growth inhibition value of the compound at 30 μM concentration is higher than 75%.

^c^Inhibition values <20% are not presented.

^d^Not determined. ^e^Cisplatin.

According to the substituents on C-16, the tested twelve phosphine oxide derivatives can be divided into three main groups. The di(naphthalen-2-yl) analogues are **17**‒**20**. Compounds **17** and **18** are 3-methoxy derivatives differing in the orientation of their C-16 substituents. Although these compounds exerted negligible growth inhibitory effect on most of the tested cell lines, they displayed the strongest effect against UPCI-SCC-131 cells. The other two substances are 3-benzyloxy derivatives, substituted at the 16α (**19**) or 16β (**20**) position. Their cell growth-inhibitory capacity was demonstrated to be more pronounced than that of their 3-methoxy pairs. Both benzyl ethers (**19** and **20**) inhibited cell division with the highest activity on T47D breast cancer cells. None of them was able to exhibit significant inhibitory effect on cell proliferation of MDA-MB-231 cells.

In a similar manner, the group of di-*para*-tolyl analogues contains two 3-benzyloxy (**15** and **16**) and two 3-methoxy (**13** and **14**) derivatives, which form epimer pairs. The benzyloxy compounds exerted moderate antiproliferative effect on the tested cell lines except SiHa, MCF-7 and MDA-MB-231 cells, where their activities were evaluated to be insignificant. Both compounds demonstrated the strongest antiproliferative activity on UPCI-SCC-131 and T47D cell lines. The 3-methoxy derivatives (**13** and **14**) belong to the four most effective phosphine oxides. The IC_50_ values of **13** and **14** are between 10 and 25 µM on all tested cell lines. Similar to the previous test compounds (**15** and **16**), these analogues possess the highest cell growth-inhibitory activity against UPCI-SCC-131 and T47D cells. They exerted the weakest effect on the proliferation of MDA-MB-231 and A2780 cells. Moreover, their tumour selectivity was also determined on mouse embryo fibroblast cells. Their IC_50_ values on non-cancerous cell lines (19.27 µM and 29.64 µM for **13** and **14**, respectively) are higher than their IC_50_ values on most of the tested cancer cell lines; therefore, these compounds possess better tumour selectivity compared to their parent compound **4**.

The third group of test compounds consists of four diphenylphosphine oxide derivatives bearing methoxy (**9** and **10**) or benzyloxy (**11** and **12**) functional groups at C-3. Between the methoxy epimers, the 16α-isomer (**9**) demonstrated significantly higher antiproliferative activity against all tested cancer cell lines compared to that of its epimer pair (**10**). Compound **9** exerted the most significant cell proliferation inhibitory effect on UPCI-SCC-131 and T47D cell lines. These IC_50_ values are in the low micromolar range like that of cisplatin. Tumour selectivity of **9** can be considered as good since its IC_50_ value determined on non-cancerous NIH/3T3 cells is four times higher than its IC_50_ value measured on UPCI-SCC-131 cells. The benzyloxy compounds (**11** and **12**) of this group also displayed significantly different antiproliferative effects against the tested cancer cell lines. Unlike the methoxy epimer pair, the benzyloxy analogue with 16β-substituent (**12**) exerted more marked inhibition on cancer cell division than its epimer (**11**). On the other point of view, **11** demonstrated the highest inhibitory values on UPCI-SCC-131 and T47D cell lines, but this pattern of antiproliferative action cannot be observed in the case of **12**. However, the IC_50_ value of **12** is 30.85 µM determined on non-cancerous NIH/3T3 cell line, its tumour selectivity is weaker than that of **9** due to its lower antiproliferative activity (IC_50_ = 13.62–29.83 µM) measured on all tested cancer cell lines.

During the selection of the utilised cancer cell lines, the HPV-status of the cell lines was taken into consideration because we wanted to compare the antiproliferative effect of the test compounds on HPV-positive and on the corresponding HPV-negative cell lines. Among the tested substances, there is only a single compound (**9**), which displayed markedly different antiproliferative effect on the HPV-negative oropharyngeal cancer cells in comparison to the HPV16-positive oropharyngeal cancer cells. On the other hand, this connection has not been supported by the results measured on HPV-positive and HPV-negative cervical cancer cells. Therefore, it can be concluded that the HPV-status of the tested cancer cell lines has no substantial impact on the antiproliferative activity of the phosphine oxide derivatives of 13α-oestrone. Moreover, UPCI-SCC-131 cells seem to be the most sensitive cell line when compared to most of the tested phosphine oxide derivatives.

In summary, four phosphine oxide derivatives (**9**, **12**–**14**) of the twelve newly synthesised 13α-oestrone analogues modified at the A- and D-ring have been identified as promising cell proliferation inhibiting agents. Their antiproliferative activity proved to be lower than that of their parent compounds. However, their tumour selectivity was better due to the modification of their chemical structure. Outstanding cell proliferation inhibitory activity of **9**, a diphenylphosphine oxide analogue, has been revealed on UPCI-SCC-131 oropharyngeal and T47D breast cancer cell lines. In both cases, the IC_50_ values of **9** and cisplatin, our positive control, are comparable. Furthermore, observation with respect to the structure–activity relationship of the tested compounds can also be determined. The UPCI-SCC-131 cells derived from oropharyngeal carcinoma and T47D cells with breast cancer origin are the most sensitive cancer cell lines to our phosphine oxide derivatives. Bulky substituents (e.g. naphthyl) in either position at C-16 eliminates the antiproliferative activity of the test compound. In contrast, the orientation of functional groups at C-16 seems to have no significant impact on cell growth-inhibitory capacity if smaller substituents (e.g. *para*-tolyl or phenyl) are present on the phosphorus atom. Finally, based on the chemical structure of the most effective diphenylphosphine oxide analogue (**9**), it can be concluded that its substituent at C-16 in α position is preferred regarding its antiproliferative activity on UPCI-SCC-131 and T47D cells. Finally, since **9**, like the other two promising compounds (**13** and **14**), belongs to the group of 3-methoxy derivatives, it suggests that 3-methoxy group can be an advantageous modification on certain 13α-oestrone derivatives.

### 3.3. Computational investigations

Considering the experimental results, computational investigations were performed for the epimer pair **9** and **10** to examine the possible energetic reason of the observed stereoselectivity. The outcome of a specific density functional calculation always gives two minimised structures, where the one with the lower total energy could be the preferred stereoisomer. Although we applied various functionals, the difference was very small between the epimers (∼ 1 kcal/mol or less), as the diastereomer with the lowest total energy was not always the same stereoisomer.

**Figure 2.**
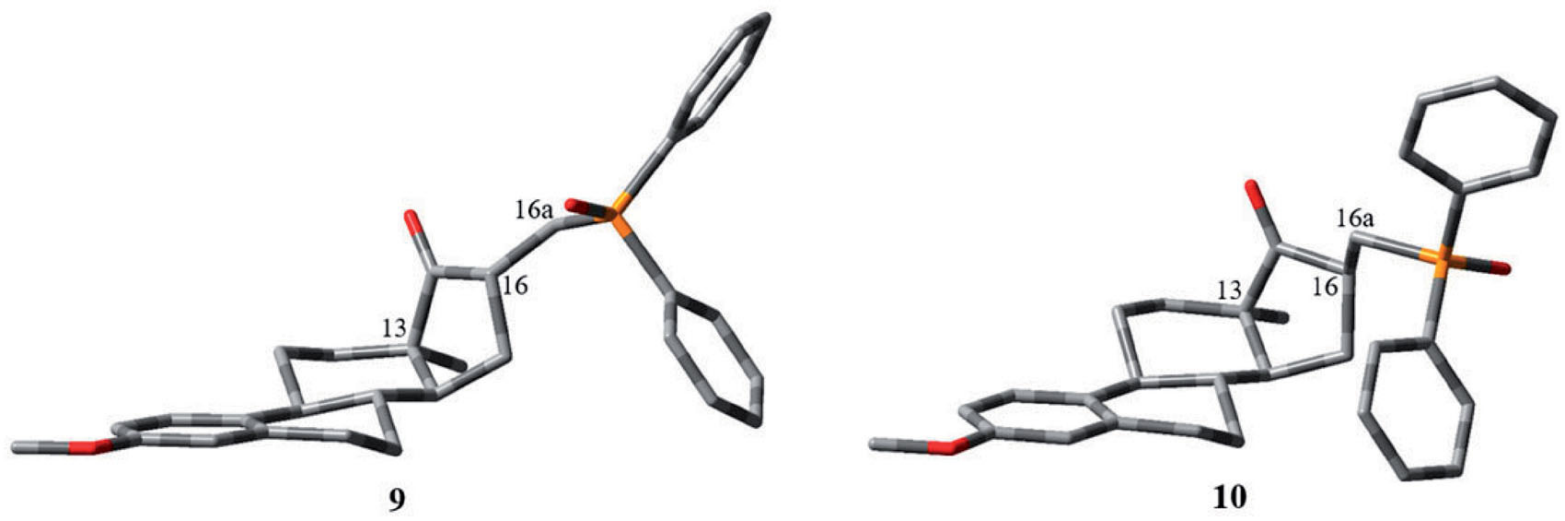
The lowest energetical conformations of the two isomers (without H-atoms) according to the BLYP-D3 calculation. The left picture shows the 16α-isomer (comp **9**), while the right one is the 16β-isomer (comp **10**). The total energies of compound **9** and **10** were –1133903.8 kcal/mol and –1133904.2 kcal/mol, respectively.

In general, simulations could not support any preference which was found in the synthetic work. Nevertheless, in Figure 2 we present the two quantum-level optimised final structures for a selected functional, namely in the BLYP-D3 case. The plausibility of the structures is demonstrated clearly by ring conformations, which are in line with the general knowledge of 13α-oestranes^28^ Namely, the A-ring is planar, the B-ring is a half-chair, while the C-ring is chair and the D-ring was found in 14β-envelope conformation.

Taking into account these results, we assume that most probably there is a reactionkinetic reason behind the diastereoselective preferences found in our study.

## 4. Conclusion

We carried out microwave-assisted phospha-Michael addition reactions in the 13α-oestrone series. Phosphorylation at C-16a resulted in two diastereomeric products (16α- and 16β) in high yields. A simple and efficient microwave-assisted methodology was elaborated for the synthesis of organophosphorus steroidal compounds representing an undervalued but promising family of potential anticancer agents in chemical space. One of the presented compounds (**9**) exhibited impressive selectivity for HPV-negative oropharyngeal cancer cell line UPCI-SCC-131 with modest action on non-cancerous fibroblasts. Our results in connection with the antiproliferative capacity of the tested compounds might underlie the importance to design and synthesise more organophosphorus steroid analogues expecting that they show higher tumour specificity and better tumour selectivity.

## Supplementary Material

Supplemental Material
