# Supplementary material for: Microwave-assisted Phospha-Michael addition reactions in the 13α-oestrone series and in vitro antiproliferative properties
Source: J Enzyme Inhib Med Chem. 2021 Aug 27;36(1):1931–7. doi: 10.1080/14756366.2021.1963241 (PMC8405091; doi:10.1080/14756366.2021.1963241)
Supplement: Supplemental Material [file IENZ_A_1963241_SM7322.zip › IENZ_1963241_SuppMat1.docx]

**Microwave-assisted Phospha-Michael Addition Reactions in the 13α-Oestrone Series and *in vitro* Antiproliferative Properties**

Erzsébet Mernyák,^1^ Sándor Bartha,^2^ Lili Kóczán,^1^ Rebeka Jójárt,^1^ Vivien Resch,^3^ Gábor Paragi,^4,5^ Máté Vágvölgyi,^6^ Attila Hunyadi,^6^ Bella Bruszel,^3^ István Zupkó,^2^ Renáta Minorics^2^*

^1^Department of Organic Chemistry, University of Szeged, Dóm tér 8, H-6720 Szeged, Hungary

^2^Department of Pharmacodynamics and Biopharmacy, University of Szeged, Eötvös u. 6, H-6720 Szeged, Hungary

^3^Department of Medicinal Chemistry, University of Szeged, Dóm tér 8, H-6720 Szeged, Hungary

^4^MTA-SZTE Biomimetic Systems Research Group, University of Szeged, Dóm tér 8, Szeged H-6720, Hungary

^5^Institute of Physics, University of Pécs, Ifjúság útja 6, Pécs H-7624, Hungary

^6^Department of Pharmacognosy, University of Szeged, Eötvös u. 6, Szeged H-6720, Hungary.

*Corresponding author. Tel.: +36 62 545567 (R. Minorics).

E-mail address: kanizsaine.minorics.renata@szte.hu (R. Minorics).

**Experimental**

**1. Chemistry**

Melting points (Mp) were determined with a Kofler hot-stage apparatus and are uncorrected. Elemental analyses were performed with a Perkin-Elmer CHN analyzer model 2400. Thin-layer chromatography: silica gel 60 F254; layer thickness 0.2 mm (Merck); eluent (ss): 50% ethyl acetate/50% dichloromethane, detection with I_2_ or UV (365 nm) after spraying with 5% phosphomolybdic acid in 50% aqueous phosphoric acid and heating at 100–120 °C for 10 min. Flash chromatography: silica gel 60, 40–63 μm (Merck). Reactions under microwave irradiation were carried out with a CEM Corporation focused microwave system, Model Discover SP. The maximum power of irradiation was 200 W. ^1^H NMR spectra were recorded in DMSO-d_6_ or CDCl_3_ solution with a Bruker DRX-500 instrument at 500 MHz, with Me_4_Si as internal standard. ^13^C NMR spectra were recorded with the same instrument at 125 MHz, and ^31^P spectra at 202 MHz under the same conditions. Mass spectrometry: full scan mass spectra of the compounds were acquired in the range of 50 to 1000 m/z with a Finnigan TSQ-7000 triple quadrupole mass spectrometer (Finnigan-MAT, San Jose, CA) equipped with a Finnigan electrospray ionization source. Analyses were performed in positive ion mode using flow injection mass spectrometry with a mobile phase of 50% aqueous acetonitrile containing 0.1 v/v% formic acid. The flow rate was 0.3 mL/min. Five µL aliquot of the samples were loaded into the flow. The ESI capillary was adjusted to 4.5 kV and N_2_ was used as a nebulizer gas. RP-HPLC separations: chromatographic method development and sample purity analysis were performed on a Phenomenex Biphenyl 250 x 4.6 mm, 5 µm column (Phenomenex Inc., Torrance, CA, USA) at 1 mL/min flow rate, while using a dual pump (PU-2080) Jasco HPLC instrument (Jasco International Co. Ltd., Hachioji, Tokyo, Japan) that was equipped with an MD-2010 Plus PDA detector to collect data in a detection range of 210–410 nm. Preparative chromatographic separations were carried out on an Armen Spot Prep II integrated HPLC purification system (Gilson, Middleton, WI, USA) with dual-wavelength detection applied at 210 and 228 nm. The separations were performed on a Phenomenex Biphenyl 250 x 21.2 mm, 5 µm column by adequately chosen eluents of methanol–water, and the flow rates were 12 mL/min.

- 1. General procedure for the phospha-Michael addition reactions

Secondary phosphine oxide (**6** or **7** or **8**, 0.5 mmol), the 3-methoxy-16-methylene-13α-oestra-1,3,5(10)-trien-17-one (**4**) (148 mg, 0.5 mmol) or the 3-benzyloxy-16-methylene-13α-oestra-1,3,5(10)-trien-17-one (**5**) (187 mg, 0.5 mmol) and acetonitrile (3 ml) were added in a 10 mL Pyrex pressure vessel (CEM, Part #: 908035) with silicone cap (CEM, Part #: 909210) and the mixture was heated in a CEM microwave reactor under stirring. After microwave irradiation, the reaction mixture was allowed to cool to room temperature. The majority of the main product was filtered as a white precipitate. The filtrate was evaporated in vacuo, the obtained product mixture was purified by flash chromatography, and subsequently, the corresponding diastereomeric products were separated by preparative RP-HPLC.

1.1.1. Synthesis of [(3-methoxy-17-oxo-13α-oestra-1,3,5(10)-trien-16α-yl)methyl]diphenylphosphine oxide (**9**) and [(3-methoxy-17-oxo-13α-oestra-1,3,5(10)-trien-16β-yl)methyl]diphenylphosphine oxide (**10**)

As described in Section 1.1., ketone **4** (148 mg, 0.5 mmol) was reacted with diphenylphosphine oxide **6** (101 mg, 0.5 mmol). Compound **9** was obtained as a white precipitate (98 mg, 39%). The crude mixture remained from the filtrate was purified by flash chromatography with EtOAc–CH_2_Cl_2_ (10:90, v/v) as eluent that resulted in the isolation of an enriched mixture of products **9** and **10** (120 mg, 48%). Subsequently, the diastereomers were separated by means of preparative RP-HPLC with an isocratic ratio of MeOH–H_2_O (88:12, v/v) as eluent. The first-eluted compound **9** was obtained as a white solid after evaporation of the eluent (76 mg, 30%), mp 200‒201 °C, R_f_ = 0.52; Anal. Calcd. for C_32_H_35_O_3_P: C, 77.09; H, 7.08. Found: C, 77.16; H, 7.01. ^1^H NMR (CDCl_3_) δ ppm: 1.00 (s, 3H, H-18), 2.76 (m, 2H, H-6), 3.08 (m, 1H), 3.75 (s, 3H, OCH_3_), 6.58 (d, 1H, *J*= 2.6 Hz, H-4), 6.67 (dd, 1H, *J*= 2.6 Hz, *J*= 8.6 Hz, H-2), 7.13 (d, 1H, *J*= 8.6 Hz, H-1), 7.45–7.54 (overlapping multiplets, 6H), 7.73–7.80 (overlapping multiplets, 4H). ^13^C NMR (CDCl_3_) δ ppm: 25.8 (C-18), 28.4 (CH_2_), 30.2 (CH_2_), 30.3 (CH_2_), 32.8 (CH_2_), 32.9 (d, *J*= 73.0 Hz, C-16a), 40.1 (d, *J*= 3.6 Hz, C-16), 41.4 (CH), 41.6 (CH), 48.1 (CH), 49.9 (C-13), 55.2 (OCH_3_), 111.8 (CH), 113.5 (CH), 126.8 (CH), 128.6–128.9 (overlapping multiplets, 4C, 4xCH), 130.6 (d, *J*= 8.9 Hz, 2xCH), 130.9 (d, *J*= 8.9 Hz, 2xCH), 131.7 (C-10), 131.9 (d, *J*= 98.6 Hz, C), 131.8 (d, *J*= 3.0 Hz, CH), 131.9 (d, *J*= 3.0 Hz, CH), 133.7 (d, *J*= 99.5 Hz, C), 138.1 (C-5), 157.5 (C-3), 222.2 (d, *J*= 13.4 Hz, C-17). ^31^P NMR (CDCl_3_) δ ppm: 31.3. MS *m/z* (%): 499 (100, [M+H]^+^).

Continued elution yielded compound **10** (38 mg, 15%). mp 158‒159 °C, R_f_ = 0.44; Anal. Calcd. for C_32_H_35_O_3_P: C, 77.09; H, 7.08. Found: C, 77.17; H, 7.00. ^1^H NMR (CDCl_3_) δ ppm: 1.08 (s, 3H, H-18), 2.70–2.84 (overlapping multiplets, 3H, H-6 and H-16), 3.06 (m, 1H), 3.76 (s, 3H, OCH_3_), 6.60 (d, 1H, *J*= 2.6 Hz, H-4), 6.70 (dd, 1H, *J*= 2.6 Hz, *J*= 8.6 Hz, H-2), 7.15 (d, 1H, *J*= 8.6 Hz, H-1), 7.45–7.53 (overlapping multiplets, 6H), 7.71–7.80 (overlapping multiplets, 4H). ^13^C NMR (CDCl_3_) δ ppm: 25.7 (C-18), 27.7 (CH_2_), 27.8 (CH_2_), 28.4 (CH_2_); 30.4 (CH_2_), 30.8 (CH_2_); 32.7 (d, *J*= 73.4 Hz, C-16a), 37.3 (d, *J*= 3.6 Hz, C-16), 40.8 (CH), 44.0 (CH), 48.1 (CH), 49.3 (C-13), 55.2 (OCH_3_), 112.0 (C-4), 113.6 (C-2), 127.0 (C-1), 128.6–128.9 (overlapping multiplets, 4C, 4xCH), 130.6 (d, *J*= 8.9 Hz, 2xCH), 130.9 (d, *J*= 8.9 Hz, 2xCH), 131.8 (d, *J*= 3.0 Hz, CH), 131.9 (d, *J*= 3.0 Hz, CH), 132.1 (C-10), 132.6 (d, *J*= 98.6 Hz, C), 134.0 (d, *J*= 99.5 Hz, C), 137.9 (C-5), 157.6 (C-3), 220.7 (d, *J*= 13.4 Hz, C-17). ^31^P NMR (CDCl_3_) δ ppm: 31.0. MS *m/z* (%): 499 (100, [M+H]^+^).

1.1.2. Synthesis of [(3-benzyloxy-17-oxo-13α-oestra-1,3,5(10)-trien-16α-yl)methyl]diphenylphosphine oxide (**11**) and [(3-benzyloxy-17-oxo-13α-oestra-1,3,5(10)-trien-16β-yl)methyl]diphenylphosphine oxide (**12**)

As described in Section 1.1., ketone **5** (186 mg, 0.5 mmol) was reacted with diphenylphosphine oxide **6** (101 mg, 0.5 mmol). Compound **11** was obtained as a white precipitate (120 mg, 42%). The crude mixture remained from the filtrate was purified by flash chromatography with EtOAc–CH_2_Cl_2_ (10:90, v/v) as eluent that allowed us to isolate an enriched mixture of compounds **11** and **12** (140 mg, 49%). Subsequently, the obtained diastereomers were separated by means of preparative RP-HPLC with MeOH–H_2_O (91:9, v/v) as eluent. The first-eluted compound **11** was obtained as a white solid after evaporation of the eluent (58 mg, 20%), mp 182‒183 °C, R_f_ = 0.60; Anal. Calcd. for C_38_H_39_O_3_P: C, 79.42; H, 6.84. Found: C, 79.51; H, 6.76. ^1^H NMR (CDCl_3_) δ ppm: 1.00 (s, 3H, H-18), 2.75 (m, 2H, H-6), 3.08 (m, 1H), 5.01 (s, 2H, OCH_2_), 6.67 (d, 1H, *J*= 2.5 Hz, H-4), 6.74 (dd, *J*= 2.5 Hz, *J*= 8.6 Hz, H-2), 7.13 (d, 1H, *J*= 8.6 Hz, H-1), 7.30 (m, 1H), 7.35–7.41 (overlapping multiplets, 4H), 7.46–7.52 (overlapping multiplets, 6H), 7.74–7.80 (overlapping multiplets, 4H). ^13^C NMR (CDCl_3_) δ ppm: 25.8 (C-18), 28.3 (CH_2_), 28.4 (CH_2_), 30.2 (CH_2_), 30.3 (CH_2_), 32.8 (CH_2_), 32.9 (d, *J*= 73.2 Hz, C-16a), 40.1 (d, *J*= 3.6 Hz, C-16), 41.4 (CH), 41.6 (CH), 48.0 (CH), 50.0 (C-13), 69.9 (OCH_2_), 112.6 (CH), 114.6 (CH), 126.8 (CH), 127.4 (2xCH), 127.8 (CH), 128.5 (2xCH), 128.6–128.9 (overlapping multiplets, 4C, 4xCH), 130.6 (d, *J*= 8.9 Hz, 2xCH), 130.9 (d, *J*= 8.9 Hz, 2xCH), 131.7 (d, *J*= 8.9, CH), 131.8 (d, *J*= 98.6 Hz, C), 131.9 (C-10), 132.0 (d, *J*= 8.9 Hz, CH), 133.8 (d, *J*= 99.5 Hz, C), 137.2 (C), 138.1 (C-5), 156.8 (C-3), 222.1 (d, *J*= 13.4 Hz, C-17).^31^P NMR (CDCl_3_) δ ppm: 31.3. MS *m/z* (%): 575 (100, [M+H]^+^).

Continued elution yielded compound **12** (76 mg, 27%). mp 157‒158 °C, R_f_ = 0.48; C_38_H_39_O_3_P: C, 79.42; H, 6.84. Found: C, 79.50; H, 6.74. ^1^H NMR (CDCl_3_) δ ppm: 1.08 (s, 3H, H-18), 2.68–2.84 (overlapping multiplets, 3H, H-6 and H-16), 3.06 (m, H-16a), 5.02 (s, 2H, OCH_2_), 6.67 (d, 1H, *J* = 2.6 Hz, H-4), 6.77 (dd, 1H, *J*= 2.6 Hz, *J*= 8.6 Hz, H-2), 7.14 (d, 1H, *J*= 8.6 Hz, H-1), 7.30 (t, 1H, *J* = 7.3 Hz); 7.37 (t, 2H, *J* = 7.3 Hz); 7.41 (t, 2H, *J* = 7.3 Hz); 7.45–7.53 (overlapping multiplets, 6H), 7.72–7.80 (overlapping multiplets, 4H). ^13^C NMR (CDCl_3_) δ ppm: 25.7 (C-18), 27.7 (CH_2_), 27.8 (CH_2_), 28.4 (CH_2_); 30.4 (CH_2_), 30.8 (CH_2_); 32.7 (d, *J*= 73.4 Hz, C-16a), 37.4 (d, *J*= 3.6 Hz, C-16), 40.8 (CH), 44.0 (CH), 48.1 (CH), 49.3 (C-13), 70.0 (OCH_2_), 112.8 (C-4), 114.6 (C-2), 127.0 (CH), 127.4 (2xCH), 127.8 (CH); 128.5 (2C); 128.6–128.9 (overlapping multiplets, 4C, 4xCH), 130.7 (d, *J*= 8.9 Hz, 2xCH), 130.9 (d, *J*= 8.9 Hz, 2xCH), 131.7 (d, *J*= 3.0 Hz, CH), 131.8 (d, *J*= 3.0 Hz, CH), 132.4 (C-10), 132.6 (d, *J*= 98.6 Hz, C), 134.0 (d, *J*= 99.5 Hz, C), 137.4 (C); 138.0 (C-5), 156.9 (C-3), 220.7 (d, *J*= 13.4 Hz, C-17). ^31^P NMR (CDCl_3_) δ ppm: 31.0. MS *m/z* (%): 575 (100, [M+H]^+^).

1.1.3. Synthesis of [(3-methoxy-17-oxo-13α-oestra-1,3,5(10)-trien-16α-yl)methyl]di-*p*-tolylphosphine oxide (**13**) and [(3-methoxy-17-oxo-13α-oestra-1,3,5(10)-trien-16β-yl)methyl]di-*p*-tolylphosphine oxide (**14**)

As described in Section 1.1., ketone **4** (148 mg, 0.5 mmol) was reacted with di-*p*-tolylphosphine oxide **7** (115 mg, 0.5 mmol). Compound **13** was obtained as a white precipitate (110 mg, 42%). The crude mixture remained from the filtrate was purified by flash chromatography with EtOAc–CH_2_Cl_2_ (10:90, v/v) as eluent that allowed us the isolation of an enriched mixture of products **13** and **14** (127 mg, 48%). Subsequently, the obtained diastereomers were separated by means of preparative RP-HPLC with MeOH–H_2_O (91:9, v/v) as eluent. The first-eluted compound **13** was obtained as a white solid after evaporation of the eluent (89 mg, 34%), mp 209‒210 °C, R_f_ = 0.58; Anal. Calcd. for C_34_H_39_O_3_P: C, 77.54; H, 7.46. Found: C, 77.66; H, 7.35. ^1^H NMR (CDCl_3_) δ ppm: 0.99 (s, 3H, H-18), 2.36 and 2.38 (2xs, 2x3H, 2xCH_3_), 2.77 (m, 2H, H-6), 3.02 (m, 1H), 3.75 (s, 3H, OCH_3_), 6.58 (d, 1H, *J*= 2.5 Hz, H-4), 6.67 (dd, *J*= 2.5 Hz, *J*= 8.6 Hz, H-2), 7.13 (d, 1H, *J*= 8.6 Hz, H-1), 7.25 (m, 4H), 7.60–7.66 (overlapping multiplets, 4H). ^13^C NMR (CDCl_3_) δ ppm: 21.5 (2C, 2xCH_3_), 25.7 (C-18), 28.3 (CH_2_), 28.4 (CH_2_), 30.2 (CH_2_), 30.3 (CH_2_), 32.8 (CH_2_), 32.9 (d, *J*= 73.2 Hz, C-16a), 40.2 (d, *J*= 3.6 Hz, C-16), 41.4 (CH), 41.6 (CH), 48.0 (CH), 49.9 (C-13), 55.2 (OCH_3_), 111.8 (CH), 113.5 (CH), 126.8 (CH), 128.7 (d, *J*= 98.6 Hz, C), 129.3–129.6 (overlapping multiplets, 4C, 4xCH), 130.6 (d, *J*= 98.6 Hz, C), 130.7 (d, *J*= 9.7 Hz, 2xCH), 130.9 (d, *J*= 9.7 Hz, 2xCH), 131.7 (C-10), 138.1 (C-5), 142.2 (C), 142.4 (C), 157.5 (C-3), 222.3 (d, *J*= 14.0 Hz, C-17). ^31^P NMR (CDCl_3_) δ ppm: 31.7. MS *m/z* (%): 527 (100, [M+H]^+^).

Continued elution yielded compound **14** (28 mg, 11%). mp 162‒163 °C, R_f_ = 0.50; Anal. Calcd. for C_34_H_39_O_3_P: C, 77.54; H, 7.46. Found: C, 77.64; H, 7.37. ^1^H NMR (CDCl_3_) δ ppm: 1.07 (s, 3H, H-18), 2.38 and 2.39 (2xs, 2x3H, 2xCH_3_), 2.51 (m, 1H, H-16a), 2.69–2.82 (overlapping multiplets, 3H, H-6 and H-16), 3.01 (m, 1H, H-16a), 3.76 (s, 3H, 3-OCH_3_), 6.59 (d, 1H, *J*= 2.7 Hz, H-4), 6.70 (dd, *J*= 2.7 Hz, *J*= 8.6 Hz, H-2), 7.15 (d, 1H, *J*= 8.6 Hz, H-1), 7.25–7.27 (overlapping multiplets, 4H), 7.59–7.67 (overlapping multiplets, 4H). ^13^C NMR (CDCl_3_) δ ppm: 21.5 (2C, 2xCH_3_), 25.7 (C-18), 27.7 (CH_2_), 27.8 (CH_2_), 28.4 (CH_2_); 30.4 (CH_2_), 30.9 (CH_2_); 32.9 (d, *J*= 73.4 Hz, C-16a), 37.4 (d, *J*= 3.6 Hz, C-16), 40.8 (CH), 44.0 (CH), 48.2 (CH), 49.3 (C-13), 55.2 (OCH_3_), 112.0 (C-4), 113.6 (C-2), 127.0 (C-1), 129.3–129.5 (overlapping multiplets, 4C, 4xCH), 129.5 (d, *J*= 98.6 Hz, C), 130.6 (d, *J*= 8.9 Hz, 2xCH), 130.6 (d, *J*= 8.9 Hz, 2xCH), 130.9 (d, *J*= 99.5 Hz, C), 132.1 (C-10), 137.9 (C-5), 142.1 (C), 142.2 (C), 157.6 (C-3), 220.9 (d, *J*= 13.4 Hz, C-17). ^31^P NMR (CDCl_3_) δ ppm: 31.4. MS *m/z* (%): 527 (100, [M+H]^+^).

1.1.4. Synthesis of [(3-benzyloxy-17-oxo-13α-oestra-1,3,5(10)-trien-16α-yl)methyl]di-*p*-tolylphosphine oxide (**15**) and [(3-benzyloxy-17-oxo-13α-oestra-1,3,5(10)-trien-16β-yl)methyl]di-*p*-tolylphosphine oxide (**16**)

As described in Section 1.1., ketone **5** (186 mg, 0.5 mmol) was reacted with di-*p*-tolylphosphine oxide **7** (115 mg, 0.5 mmol). Compound **15** was obtained as a white precipitate (125 mg, 42%). The crude mixture remained from the filtrate was purified by flash chromatography with EtOAc–CH_2_Cl_2_ (10:90, v/v) as eluent that resulted in the isolation of an enriched mixture of products **15** and **16** (141 mg, 47%). Subsequently, the diastereomers were separated by means of preparative RP-HPLC with MeOH–H_2_O (92:8, v/v) as eluent. The first-eluted compound **15** was obtained as a white solid after evaporation of the eluent (83 mg, 28%), mp 207‒208 °C, R_f_ = 0.68; Anal. Calcd. for C_40_H_43_O_3_P: C, 79.71; H, 7.19. Found: C, 79.84; H, 7.09. ^1^H NMR (CDCl_3_) δ ppm: 0.99 (s, 3H, H-18), 2.36 and 2.39 (2xs, 2x3H, 2xCH_3_), 2.75(m, 2H, H-6), 3.03 (m, 1H), 5.01 (s, 2H, OCH_2_), 6.67 (d, 1H, *J*= 2.1 Hz, H-4), 6.74 (dd, *J*= 2.1 Hz, *J*= 8.5 Hz, H-2), 7.12 (d, 1H, *J*= 8.5 Hz, H-1), 7.25–7.27 (overlapping multiplets, 4H), 7.30 (m, 1H), 7.37 (m, 2H), 7.40 (m, 2H), 7.60–7.66 (overlapping multiplets, 4H). ^13^C NMR (CDCl_3_) δ ppm: 21.5 (2C, 2xCH_3_), 25.8 (C-18), 28.3 (CH_2_), 28.4 (CH_2_), 30.2 (CH_2_), 30.3 (CH_2_), 32.8 (CH_2_), 32.9 (d, *J*= 73.2 Hz, C-16a), 40.1 (d, *J*= 3.6 Hz, C-16), 41.4 (CH), 41.6 (CH), 48.0 (CH), 50.0 (C-13), 69.9 (OCH_2_), 112.6 (CH), 114.6 (CH), 126.8 (CH), 127.4 (2xCH), 127.8 (CH), 128.5 (2xCH), 128.9 (d, *J*= 99.5 Hz, C), 129.3–129.6 (overlapping multiplets, 4C, 4xCH), 130.6 (d, *J*= 8.9 Hz, 2xCH), 130.7 (d, *J*= 99.5 Hz, C), 130.9 (d, *J*= 8.9 Hz, 2xCH), 132.0 (C-10), 137.2 (C), 138.2 (C-5), 142.1 (C), 142.3 (C), 156.8 (C-3), 222.1 (d, *J*= 13.4 Hz, C-17). ^31^P NMR (CDCl_3_) δ ppm: 31.6. MS *m/z* (%): 603 (100, [M+H]^+^).

Continued elution yielded compound **16** (43 mg, 14%). mp 152‒153 °C, R_f_ = 0.54; Anal. Calcd. for C_40_H_43_O_3_P: C, 79.71; H, 7.19. Found: C, 79.82; H, 7.10. ^1^H NMR (CDCl_3_) δ ppm: 1.07 (s, 3H, H-18), 2.38 and 2.39 (2xs, 2x3H, 2xCH_3_), 2.51 (m, 1H, H-16a), 2.69–2.82 (overlapping multiplets, 3H, H-6 and H-16), 3.01 (m, 1H, H-16a), 5.03 (s, 2H, OCH_2_), 6.68 (d, 1H, *J*= 2.7 Hz, H-4), 6.77 (dd, *J*= 2.7 Hz, *J*= 8.6 Hz, H-2), 7.15 (d, 1H, *J*= 8.6 Hz, H-1), 7.25–7.28 (overlapping multiplets, 4H), 7.32 (m, 1H), 7.37 (m, 2H), 7.42 (m, 2H), 7.59–7.67 (overlapping multiplets, 4H). ^13^C NMR (CDCl_3_) δ ppm: 21.5 (2C, 2xCH_3_), 25.7 (C-18), 27.7 (CH_2_), 27.8 (CH_2_), 28.4 (CH_2_); 30.4 (CH_2_), 30.9 (CH_2_); 32.9 (d, *J*= 73.4 Hz, C-16a), 37.4 (d, *J*= 3.6 Hz, C-16), 40.8 (CH), 44.0 (CH), 48.2 (CH), 49.3 (C-13), 70.0 (OCH_2_), 112.8 (C-4), 114.6 (C-2), 127.1 (C-1), 127.4 (2xCH), 127.8 (CH), 128.5 (2xCH), 129.3–129.5 (overlapping multiplets, 4C, 4xCH), 129.5 (d, *J*= 98.6 Hz, C), 130.6 (d, *J*= 8.9 Hz, 2xCH), 130.9 (d, *J*= 8.9 Hz, 2xCH), 130.9 (d, *J*= 99.5 Hz, C), 132.4 (C-10), 137.4 (C), 138.0 (C-5), 142.1 (C), 142.2 (C), 156.9 (C-3), 220.8 (d, *J*= 13.4 Hz, C-17). ^31^P NMR (CDCl_3_) δ ppm: 31.4. MS *m/z* (%): 603 (100, [M+H]^+^).

1.1.5. Synthesis of [(3-methoxy-17-oxo-13α-oestra-1,3,5(10)-trien-16α-yl)methyl]di(naphtalen-2-yl)phosphine oxide (**17**) and [(3-methoxy-17-oxo-13α-oestra-1,3,5(10)-trien-16β-yl)methyl]di(naphtalen-2-yl)phosphine oxide (**18**)

As described in Section 1.1., ketone **4** (148 mg, 0.5 mmol) was reacted with di(naphtalen-2-yl)phosphine oxide **8** (115 mg, 0.5 mmol). Compound **17** was obtained as a white precipitate (131 mg, 44%). The crude mixture remained from the filtrate was purified by flash chromatography with EtOAc–CH_2_Cl_2_ (10:90, v/v) as eluent that allowed us to isolate an enriched mixture of products **17** and **18** (145 mg, 48%). Subsequently, the obtained diastereomers were separated by means of preparative RP-HPLC with MeOH–H_2_O (95:5, v/v) as eluent. The first-eluted compound **17** was obtained as a white solid after evaporation of the eluent (90 mg, 30%), mp 200‒201 °C, R_f_ = 0.73; Anal. Calcd. for C_40_H_39_O_3_P: C, 80.24; H, 6.57. Found: C, 80.32; H, 6.50. ^1^H NMR (CDCl_3_) δ ppm: 1.02 (s, 3H, H-18), 2.59 and 2.71 (2xm, 2x1H, H-6), 3.30 (m, 1H), 3.73 (s, 3H, OCH_3_), 6.53 (d, 1H, *J*= 2.6 Hz, H-4), 6.63 (dd, 1H, *J*= 2.6 Hz, *J*= 8.6 Hz, H-2), 7.10 (d, 1H, *J*= 8.6 Hz, H-1), 7.54–7.60 (overlapping multiplets, 4H), 7.71–7.76 (overlapping multiplets, 2H), 7.85 (t, *J*= 8.1 Hz, 2H), 7.90–7.94 (overlapping multiplets, 4H), 8.42–8.49 (overlapping multiplets, 2H). ^13^C NMR (CDCl_3_) δ ppm: 25.8 (C-18), 28.3 (CH_2_), 28.4 (CH_2_), 30.1 (CH_2_), 30.3 (CH_2_), 32.8 (CH_2_), 32.9 (d, *J*= 73.0 Hz, C-16a), 40.3 (d, *J*= 3.6 Hz, C-16), 41.3 (CH), 41.6 (CH), 48.1 (CH), 50.0 (C-13), 55.1 (OCH_3_), 111.7 (CH), 113.5 (CH), 125.5–125.6 (overlapping multiplets, 2C, 2xCH), 126.7 (C-1), 127.0 (2C, 2xCH), 127.8 (d, 2C, *J*= 4.0 Hz, 2xCH), 127.8 (d, 2C, *J*= 4.0 Hz, 2xCH), 128.6 (d, *J*= 11.2 Hz, CH), 128.8 (d, *J*= 11.2 Hz, CH), 128.9 (2C, 2xCH), 128.9 (d, *J*= 99.5 Hz, C), 130.7 (d, *J*= 99.5 Hz, C), 131.6 (C-10), 132.5 (d, *J*= 8.5 Hz, CH), 132.5–132.7 (overlapping multiplets, 2xC), 133.0 (d, *J*= 8.5 Hz, CH), 134.7 (d, *J*= 2.0 Hz, C), 134.8 (d, *J*= 2.0 Hz, C), 138.0 (C-5), 157.5 (C-3), 222.1 (d, *J*= 13.4 Hz, C-17). ^31^P NMR (CDCl_3_) δ ppm: 31.6. MS *m/z* (%): 599 (100, [M+H]^+^).

Continued elution yielded compound **18** (41 mg, 14%). mp 142‒143 °C, R_f_ = 0.65; Anal. Calcd. for C_40_H_39_O_3_P: C, 80.24; H, 6.57. Found: C, 80.33; H, 6.49. ^1^H NMR (CDCl_3_) δ ppm: 1.06 (s, 3H, H-18), 2.74 (m, 2H, H-6), 2.87 (m, 1H, H-16), 3.28 (m, 1H, H-16a), 3.75 (s, 3H, 3-OCH_3_), 6.58 (d, 1H, *J*= 2.0 Hz, H-4), 6.69 (dd, 1H, *J*= 2.0 Hz, *J*= 8.5 Hz, H-2), 7.15 (d, 1H, *J*= 8,5 Hz, H-1), 7.54–7.61 (overlapping multiplets, 4H), 7.70 (t, 1H, *J*= 9.7 Hz), 7.75 (t, 1H, *J*= 9.7 Hz), 7.85–7.95 (overlapping multiplets, 6H), 8.45 (t, 2H, *J*= 14.2 Hz). ^13^C NMR (CDCl_3_) δ ppm: 25.7 (C-18), 27.7 (CH_2_), 27.8 (CH_2_), 28.5 (CH_2_), 30.3 (CH_2_), 30.9 (CH_2_), 32.6 (d, *J*= 73.4 Hz, C-16a), 37.4 (d, *J*= 3.8 Hz, C-16), 40.8 (CH), 44.1 (CH), 48.1 (CH), 49.3 (C-13), 55.2 (3-OCH_3_), 112.0 (C-4), 113.6 (C-2), 125.5–125.7 (overlapping multiplets, 2C, 2xCH), 127.0 (3C, 3xCH), 127.9 (2C, 2xCH), 128.2 (2C, 2xCH), 128.5–129.0 (overlapping multiplets, 4C, 4xCH), 129.6 (d, *J*= 100.1 Hz, CH), 131.0 (d, *J*= 100.1 Hz, CH), 132.1 (C-10), 132.6 (d, *J*= 10.4 Hz, CH), 132.7 (C), 132.8 (C), 133.0 (d, *J*= 10.4 Hz, CH), 134.7 (C), 134.8 (C), 137.9 (C), 157.6 (C-3), 220.8 (d, *J*= 13.4 Hz, C-17). ^31^P NMR (CDCl_3_) δ ppm: 31.4. MS *m/z* (%): 599 (100, [M+H]^+^).

1.1.6. Synthesis of [(3-benzyloxy-17-oxo-13α-oestra-1,3,5(10)-trien-16α-yl)methyl]di(naphtalen-2-yl)phosphine oxide (**19**) and [(3-benzyloxy-17-oxo-13α-oestra-1,3,5(10)-trien-16β-yl)methyl]di(naphtalen-2-yl)phosphine oxide (**20**)

As described in Section 1.1., ketone **5** (186 mg, 0.5 mmol) was reacted with di(naphtalen-2-yl)phosphine oxide **8** (151 mg, 0.5 mmol). Compound **19** was obtained as a white precipitate (130 mg, 39%). The crude mixture remained from the filtrate was purified by flash chromatography with EtOAc–CH_2_Cl_2_ (10:90, v/v) as eluent that allowed us the isolation of an enriched mixture of products **19** and **20** (159 mg, 48%). Subsequently, the obtained diastereomers were separated by means of preparative RP-HPLC with MeOH–H_2_O (95:5, v/v) as eluent. The first-eluted compound **19** was obtained as a white solid after evaporation of the eluent (95 mg, 29%), mp 180‒181 °C, R_f_ = 0.77; Anal. Calcd. for C_46_H_43_O_3_P: C, 81.87; H, 6.42. Found: C, 81.92; H, 6.36. ^1^H NMR (CDCl_3_) δ ppm: 1.02 (s, 3H, H-18), 2.59 and 2.68 (2xm, 2x1H, H-6), 3.29 (m, 1H), 4.98 (s, 2H, OCH_2_), 6.60 (d, 1H, *J*= 2.5 Hz, H-4), 6.71 (dd, 1H, *J*= 2.5 Hz, *J*= 8.6 Hz, H-2), 7.10 (d, 1H, *J*= 8.6 Hz, H-1), 7.30 (t, *J*= 7.0 Hz, 1H), 7.34–7.40 (overlapping multiplets, 4H), 7.54–7.61 (overlapping multiplets, 4H), 7.71–7.77 (overlapping multiplets, 2H), 7.84–7.88 (overlapping multiplets, 2H), 7.90–7.94 (overlapping multiplets, 4H), 8.42–8.49 (overlapping multiplets, 2H). ^13^C NMR (CDCl_3_) δ ppm: 25.8 (C-18), 28.3 (CH_2_), 28.4 (CH_2_), 30.1 (CH_2_), 30.3 (CH_2_), 32.8 (CH_2_), 32.9 (d, *J*= 73.0 Hz, C-16a), 40.3 (d, *J*= 3.6 Hz, C-16), 41.3 (CH), 41.5 (CH), 48.1 (CH), 50.0 (C-13), 69.9 (OCH_2_), 112.5 (CH), 114.6 (CH), 125.5–125.6 (overlapping multiplets, 2C, 2xCH), 126.7 (C-1), 127.0 (2C, 2xCH), 127.4 (2C, 2xCH), 127.8–127.9 (overlapping multiplets, 3xCH), 128.2 (d, 2C, *J*= 11.2 Hz, 2xCH), 128.5 (2C, 2xCH), 128.6 (d, *J*= 11.2 Hz, CH), 128.9 (2C, 2xCH), 128.9 (d, *J*= 11.2 Hz, CH), 128.9 (d, *J*= 99.5 Hz, C), 130.7 (d, *J*= 99.5 Hz, C), 131.9 (C-10), 132.5 (d, *J*= 8.5 Hz, CH), 132.5–132.7 (overlapping multiplets, 2xC), 133.0 (d, *J*= 8.5 Hz, CH), 134.7 (d, *J*= 2.0 Hz, C), 134.8 (d, *J*= 2.0 Hz, C), 137.3 (C), 138.1 (C-5), 156.8 (C-3), 222.1 (d, *J*= 13.4 Hz, C-17). ^31^P NMR (CDCl_3_) δ ppm: 31.6. MS *m/z* (%): 675 (100, [M+H]^+^).

Continued elution yielded compound **20** (46 mg, 14%). mp 159‒160 °C, R_f_ = 0.71; Anal. Calcd. for C_46_H_43_O_3_P: C, 81.87; H, 6.42. Found: C, 81.94; H, 6.35. ^1^H NMR (CDCl_3_) δ ppm: 1.06 (s, 3H, H-18), 2.72 (m, 2H, H-6), 2.88 (m, 1H, H-16), 3.29 (m, 1H, H-16a), ), 5.01 (s, 2H, OCH_2_), 6.66 (d, 1H, *J*= 2.0 Hz, H-4), 6.77 (dd, 1H, *J*= 2.0 Hz, *J*= 8.5 Hz, H-2), 7.15 (d, 1H, *J*= 8,5 Hz, H-1), 7.30 (t, 1H, *J*= 7.3 Hz, 1H), 7.36 (t, 2H, *J*= 7.3 Hz, 1H), 7.40 (t, 2H, *J*= 7.3 Hz, 1H), 7.54–7.61 (overlapping multiplets, 4H), 7.70 (t, 1H, *J*= 9.7 Hz), 7.76 (t, 1H, *J*= 9.7 Hz), 7.86 (m, 2H), 7.89–7.96 (overlapping multiplets, 4H), 8.46 (t, 2H, *J*= 14.2 Hz), ^13^C NMR (CDCl_3_) δ ppm: 25.7 (C-18), 27.7 (CH_2_), 27.8 (CH_2_), 28.5 (CH_2_), 30.3 (CH_2_), 30.9 (CH_2_), 32.6 (d, *J*= 73.4 Hz, C-16a), 37.4 (d, *J*= 3.8 Hz, C-16), 40.8 (CH), 43.9 (CH), 48.1 (CH), 49.3 (C-13), 70.0 (OCH_2_), 112.8 (C-4), 114.6 (C-2), 125.5–125.7 (overlapping multiplets, 2C, 2xCH), 127.0 (3C, 3xCH), 127.4 (2C, 2xCH), 127.8 (CH), 127.9 (2C, 2xCH), 128.2 (2C, 2xCH), 128.5 (2C, 2xCH), 128.6–129.0 (overlapping multiplets, 4C, 4xCH), 129.6 (d, *J*= 100.1 Hz, CH), 131.0 (d, *J*= 100.1 Hz, CH), 132.4 (C-10), 132.6 (d, *J*= 10.4 Hz, CH), 132.7 (C), 132.8 (C), 132.9 (d, *J*= 10.4 Hz, CH), 134.7 (C), 134.8 (C), 137.4 (C), 138.0 (C), 156.9 (C-3), 220.7 (d, *J*= 13.4 Hz, C-17). ^31^P NMR (CDCl_3_) δ ppm: 31.4. MS *m/z* (%): 675 (100, [M+H]^+^).

Figure S1. The part of the HSQC spectrum of compound **20**


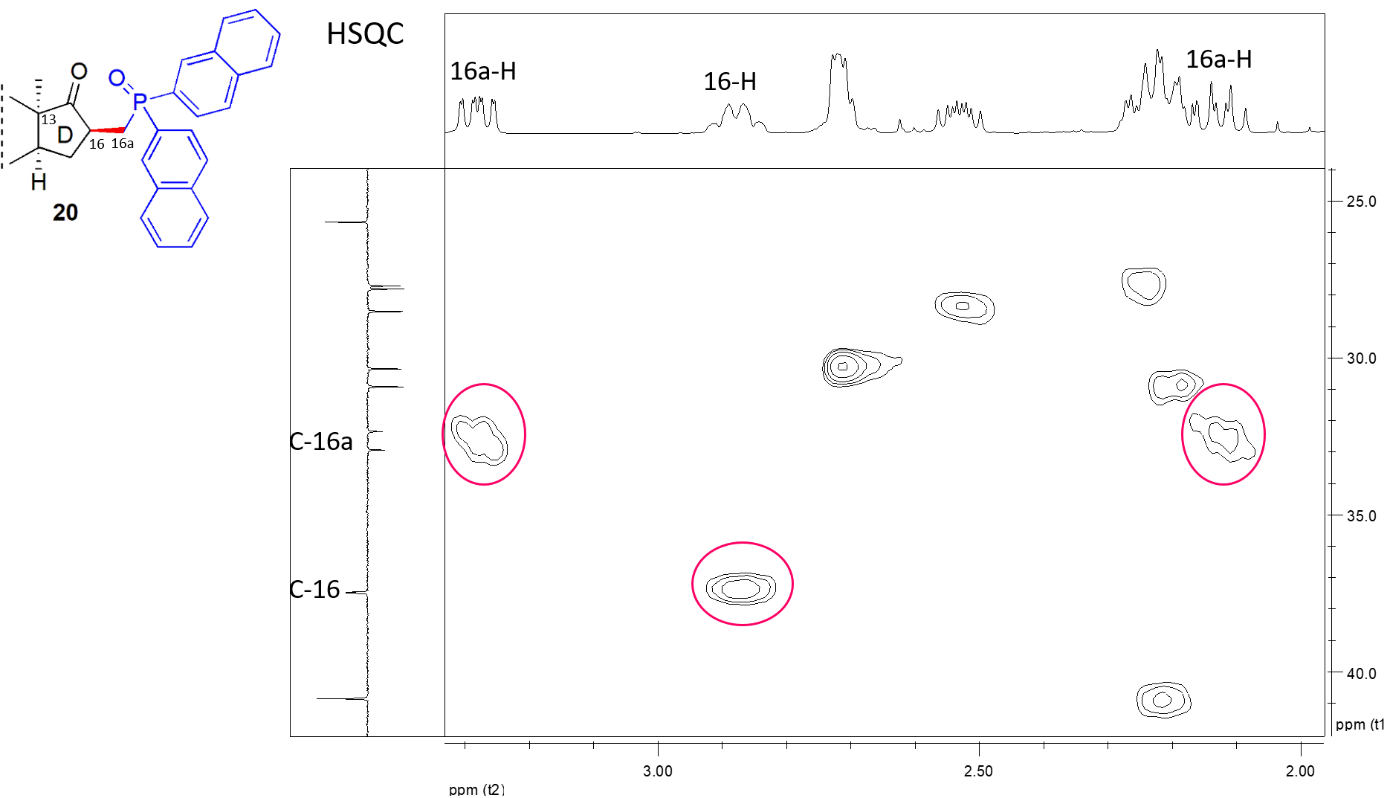


Figure S2. The part of the NOESY spectrum of compound **20**


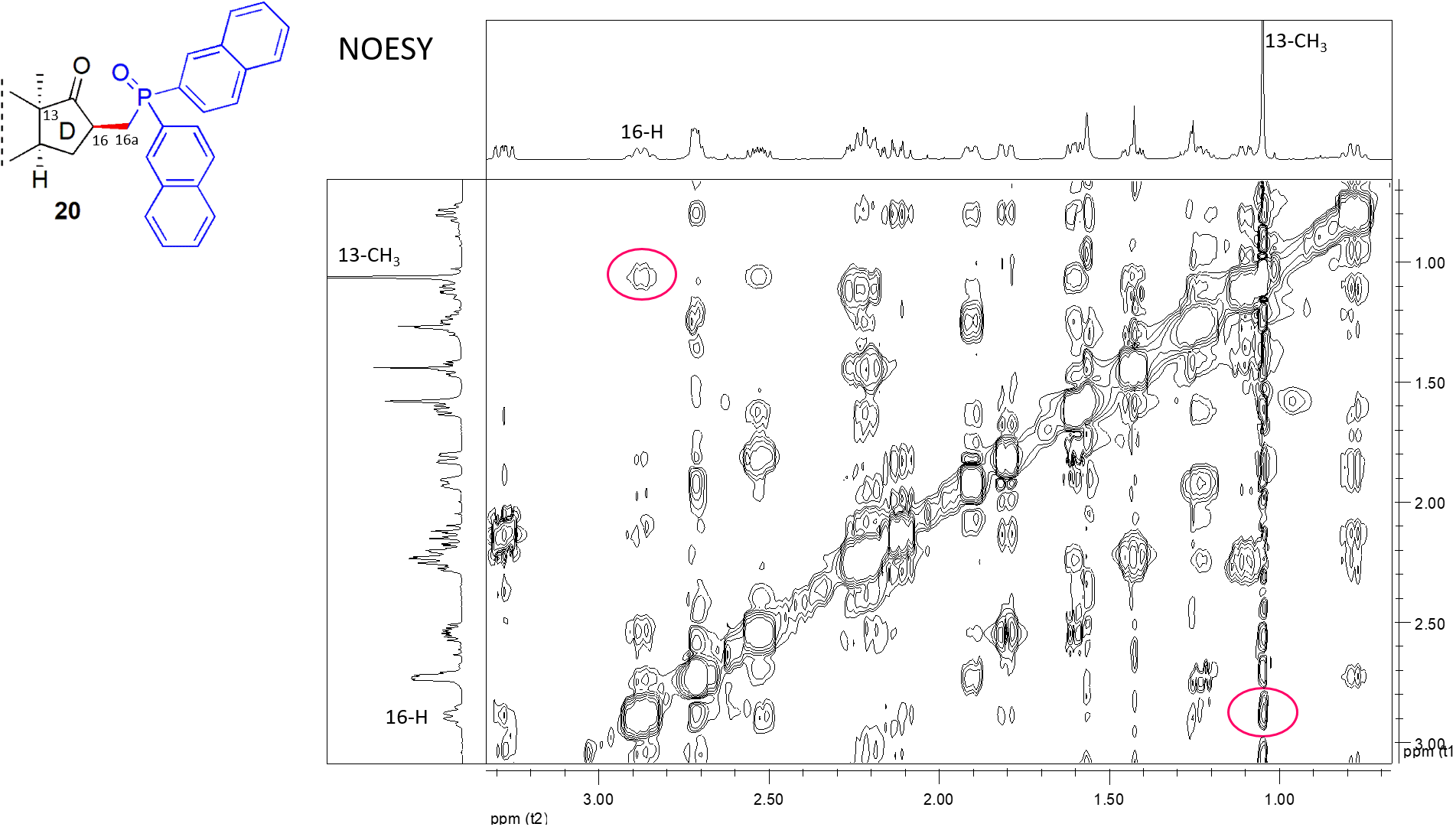


**2. Determination of antiproliferative activities**

The antiproliferative properties of the newly synthesized compounds (**9**–**20**) and their starting materials (**4** and **5**) were determined on a panel of human adherent cancer cell lines. MCF-7, MDA-MB-231 and T47D were isolated from breast cancers differing in biochemical background, while A2780 cells were isolated from ovarian cancer. HeLa and SiHa are cervical cancer cell lines positive for HPV-18 and HPV-16, respectively, while C33-A cervical cancer cells are negative for HPV. Moreover, two oropharyngeal cancer cell lines were also utilized: the HPV-negative UPCI-SCC-131 and HPV-16 positive UPCI-SCC-154. The cancer selectivity of compounds was tested on the non-cancerous mouse embryo fibroblast cell line NIH/3T3. All cell lines were purchased from European Collection of Cell Cultures (ECCAC, Salisbury, UK) exception for SiHa and C33-A (American Tissue Culture Collection, Manassas, VA, USA) and HN-SCC-131 and UPCI-SCC-154 (German Collection of Microorganisms and Cell Cultures GmbH, Braunschweig, Germany). Cells were cultivated in minimal essential medium supplemented with 10% fetal bovine serum, 1% non-essential amino acids and an antibiotic–antimycotic mixture. The medium of oropharyngeal cancer cells additionally contained 2.0 mM L-glutamine. All media and supplements were obtained from Lonza Group Ltd., Basel, Switzerland. Near-confluent cancer cells were seeded onto a 96-well microplate at a density of 5,000 cells/well, except for C33-A which were seeded at 10,000/well. After overnight standing, new medium containing the tested compounds at 10 and 30 µM final concentration was added. After incubation for 72 h at 37 °C in humidified air containing 5% CO_2_, the living cells were assayed by the addition of 20 μL of 5 mg/ml 3-(4,5-dimethylthiazol-2-yl)-2,5-diphenyltetrazolium bromide (MTT) solution. MTT was converted by intact mitochondrial reductase and precipitated as purple crystals during a 4-h contact period. The medium was next removed and the precipitated formazan crystals were dissolved in 100 μL of DMSO during a 60-min period of shaking at 37 ^о^C.

Finally, the reduced MTT was assayed at 545 nm, using a microplate reader (SPECTROstar Nano, BMG Labtech GmbH, Offenburg, Germany) utilising wells with untreated cells serving as control.^S1^ In the case of the most active compounds (i.e. higher than 75% growth inhibition at 30 µM), the assays were repeated with a set of dilutions, sigmoidal concentration–response curves were fitted to the determined data and the IC_50_ values (the concentration at which the extent of cell proliferation was half that of the untreated control) were calculated by means of GraphPad Prism 5.01 (GraphPad Software, San Diego, CA, USA). All *in vitro* experiments were carried out twice with five parallels. Stock solutions of the tested substances (10 mM) were prepared in DMSO. The highest DMSO content of the medium (0.6%) did not have any substantial effect on cell proliferation. Cisplatin (Ebewe Pharma GmbH, Unterach, Austria) was used as positive control.

**3. Computational details**

Conformal search analysis for the two stereoisomers was carried out by the Macromodel unit ^S2^ from the Schrodinger software suits^S3^ The OPLS3e^S4^ forcefield with implicit GB/SA solvation model for chloroform was applied, and the Mixed Torsional/Low-mode Sampling method was selected to sample the conformational space. For minimization, the Polak-Ribiere Conjugated Gradient (PRCG) method was chosen with increased maximal step number (5000). Following the molecular mechanical simulations, the top 10 structures were further optimized for each stereoisomer by quantum chemical calculations using the Gaussian 16 program^S5^. Density functional theory method were applied, and multiple calculations were performed with different functionals, like the MN15 and M06-2X from Truhlar’s group^S6–S7^or other popular functionals like the BLYP‑D3 and the O3LYP^S8–S11^ ones. In all the quantum level calculations the 6‑311G** gaussian basis set was applied, and the chloroform solvent was taken into account in an implicit way using the polarizable conductor calculation model (CPCM option in the SCRF keyword)^S12^. Taking the molecular mechanically minimised best 10 alpha and 10 beta geometries we optimized these structures with a selected functional. The conformations with the lowest total energy for the alpha and the beta conformers provided the quantum level optimized pairs of a specific functional, and the members of a pair were compared to each other.

**References:**

S1 Mosmann T. Rapid colorimetric assay for cellular growth and survival: Application to proliferation and cytotoxicity assays. Journal of immunological methods. 1983, 65, 55–63

S2 MacroModel, S., Schrödinger Release 2020-4, Schrödinger LLC, New York, NY, 2020. 2020.

S3 Schrödinger, Schrödinger Release 2020-4, LLC, New York, NY, 2020. 2020.

S4 Harder, E.; Damm, W.; Maple, J.; Wu, C.; Reboul, M.; Xiang, J. Y.; Wang, L.; Lupyan, D.; Dahlgren, M. K.; Knight, J. L.; Kaus, J. W.; Cerutti, D. S.; Krilov, G.; Jorgensen, W. L.; Abel, R.; Friesner, R. A., OPLS3: A Force Field Providing Broad Coverage of Drug-like Small Molecules and Proteins. J Chem Theory Comput 2016, 12, 281–296.

S5 Frisch, M. J.; Trucks, G. W.; Schlegel, H. B.; Scuseria, G. E.; Robb, M. A.; Cheeseman, J. R.; Scalmani, G.; Barone, V.; Petersson, G. A.; Nakatsuji, H.; Li, X.; Caricato, M.; Marenich, A. V.; Bloino, J.; Janesko, B. G.; Gomperts, R.; Mennucci, B.; Hratchian, H. P.; Ortiz, J. V.; Izmaylov, A. F.; Sonnenberg, J. L.; Williams; Ding, F.; Lipparini, F.; Egidi, F.; Goings, J.; Peng, B.; Petrone, A.; Henderson, T.; Ranasinghe, D.; Zakrzewski, V. G.; Gao, J.; Rega, N.; Zheng, G.; Liang, W.; Hada, M.; Ehara, M.; Toyota, K.; Fukuda, R.; Hasegawa, J.; Ishida, M.; Nakajima, T.; Honda, Y.; Kitao, O.; Nakai, H.; Vreven, T.; Throssell, K.; Montgomery Jr., J. A.; Peralta, J. E.; Ogliaro, F.; Bearpark, M. J.; Heyd, J. J.; Brothers, E. N.; Kudin, K. N.; Staroverov, V. N.; Keith, T. A.; Kobayashi, R.; Normand, J.; Raghavachari, K.; Rendell, A. P.; Burant, J. C.; Iyengar, S. S.; Tomasi, J.; Cossi, M.; Millam, J. M.; Klene, M.; Adamo, C.; Cammi, R.; Ochterski, J. W.; Martin, R. L.; Morokuma, K.; Farkas, O.; Foresman, J. B.; Fox, D. J. Gaussian 16 Rev. C.01, Wallingford, CT, 2016.

S6 Yu, H. S.; He, X.; Li, S. L.; Truhlar, D. G., MN15: A Kohn-Sham global-hybrid exchange-correlation density functional with broad accuracy for multi-reference and single-reference systems and noncovalent interactions. Chem Sci 2016, 7, 5032–5051.

S7 Zhao, Y.; Truhlar, D. G., - The M06 suite of density functionals for main group thermochemistry, thermochemical kinetics, noncovalent interactions, excited states, and transition elements: two new functionals and systematic testing of four M06-class functionals and 12 other functionals. 2008, - 120 (- 1), - 241.

S8 Becke, A. D., Density-functional exchange-energy approximation with correct asymptotic behavior. Phys Rev A Gen Phys 1988, 38, 3098–3100.

S9 Lee, C.; Yang, W.; Parr, R. G., Development of the Colle-Salvetti correlation-energy formula into a functional of the electron density. Phys Rev B Condens Matter 1988, 37, 785–789.

S10 Cohen, A. J.; Handy, N. C., Dynamic correlation. Mol Phys 2001, 99 (7), 607-615.

S11 Grimme, S.; Antony, J.; Ehrlich, S.; Krieg, H., A consistent and accurate ab initio parametrization of density functional dispersion correction (DFT-D) for the 94 elements H-Pu. J Chem Phys 2010, 132, 154104.

S12 Barone, V.; Cossi, M., Quantum Calculation of Molecular Energies and Energy Gradients in Solution by a Conductor Solvent Model. J Phys Chem A 1998, 102, 2001.
